# Etoricoxib - preemptive and postoperative analgesia (EPPA) in patients with laparotomy or thoracotomy - design and protocols

**DOI:** 10.1186/1745-6215-11-66

**Published:** 2010-05-27

**Authors:** Johannes Fleckenstein, Sybille Kramer, Martin Offenbächer, Gabriel Schober, Herbert Plischke, Matthias Siebeck, Thomas Mussack, Rudolf Hatz, Lukas Lehmeyer, Philip M Lang, Bernhard Heindl, Peter Conzen, Dominik Irnich

**Affiliations:** 1 Department of Anaesthesiology, University of Munich, Germany; 2 Generation Research Project, University of Munich, Bad Tölz, Germany; 3 Department of General Surgery, Campus Innenstadt, University of Munich, Germany; 4 Department of Surgery and General Thoracic Surgery, Campus Großhadern, University of Munich, Germany; 5 Department for Anesthesia, Hawkes Bay Hospital, Hastings, New Zealand

## Abstract

**Background and Objective:**

Our objective was to report on the design and essentials of the *Etoricoxib *protocol*- Preemptive and Postoperative Analgesia (EPPA) *Trial, investigating whether preemptive analgesia with cox-2 inhibitors is more efficacious than placebo in patients who receive either laparotomy or thoracotomy.

**Design and Methods:**

The study is a 2 × 2 factorial armed, double blinded, bicentric, randomised placebo-controlled trial comparing (a) etoricoxib and (b) placebo in a pre- and postoperative setting. The total observation period is 6 months. According to a power analysis, 120 patients scheduled for abdominal or thoracic surgery will randomly be allocated to either the preemptive or the postoperative treatment group. These two groups are each divided into two arms. Preemptive group patients receive etoricoxib prior to surgery and either etoricoxib again or placebo postoperatively. Postoperative group patients receive placebo prior to surgery and either placebo again or etoricoxib after surgery (2 × 2 factorial study design). The Main Outcome Measure is the cumulative use of morphine within the first 48 hours after surgery (measured by patient controlled analgesia PCA). Secondary outcome parameters include a broad range of tests including sensoric perception and genetic polymorphisms.

**Discussion:**

The results of this study will provide information on the analgesic effectiveness of etoricoxib in preemptive analgesia and will give hints on possible preventive effects of persistent pain.

**Trial registration:**

NCT00716833

## Background

To achieve an adequate postoperative pain therapy is a medical challenge. Acute postoperative pain is followed by persistent pain in 10-50% of individuals after common operations, such as groin hernia repair, breast and thoracic surgery, leg amputation, and coronary artery bypass surgery. Since chronic pain can be severe in about 2-10% of these patients, persistent postsurgical pain represents a major, largely unrecognised clinical problem [1]. There is a discrepancy between the results of many epidemiological surveys reporting persistent pain in a significant portion of postoperative patients despite the fact that very effective analgesic tools are available to treat postoperative pain [2,3]. Postoperative analgesia improves patients rehabilitation, shortens hospital stay, and potentially decreases postoperative complications [4]. In this context, the acute pain management team responsible for the patient's analgesic therapy can play an important role in improving the patient's surgical outcomes [5,6].

Preemptive analgesia has become one of the most promising strategies of pain management [7]. The precise definition of preemptive analgesia remains controversial. However, the explanatory concept behind it indicates that an analgesic intervention begins before the noxious stimulus arises which has beneficial effects reducing postoperative pain and the occurrence of postoperative pain [7]. From a physiological view of pain, physical injury generates a complex stress response that extends beyond the nervous system contributing to the experience of postoperative pain. This response comprises of neurotransmitters, peptides, endocannabinoids, cytokines, and hormones, all of which are operating in interdependent nervous, endocrine, and immune processes to cope with the injury [8]. The transition of acute postoperative pain into a chronic pain state is a complex process that not only involves the effects of one's physiological state but also involves psychological and social-environmental factors [6,9]. All these effects are supposed to induce plasticity in spinal and supraspinal structures contributed to the chronification of postoperative pain [10,11]. Therefore, in using preemptive analgesia there is a partial stopping to some of the influencing factors experienced; which in turn, may already prevent the sensitizing effects of the surgical procedure.

Systematic reviews suggest that current preemptive analgetic therapy, e.g. systemic non-steroidal anti-inflammatory drugs (NSAIDs), decreased analgesic consumption but not postoperative pain scores [12]. This effect is more pronounced when using invasive analgesia, e.g. epidural analgesia [12,13]. Nevertheless, NSAIDs failed to elicit significant effects in all of the outcome measured values in the reviews; affected selected variables were only mentioned. Although there is a widespread belief of the efficacy of preemptive analgesia among clinicians, large scale randomised controlled trials will be necessary to prove the current concepts.

A multimodal approach which combines several agents (non-opioid analgesics, opioids, local anaesthetics) and delivery techniques (intravenous anaesthesia, patient-controlled anaesthesia, epidural and regional blocks) is currently recognised as best practice in postoperative pain management [14]. Traditional, nonspecific NSAIDs are considered an important part of postoperative pain management, resulting in improved clinical outcomes. Otherwise their role is limited in the peri- and postoperative setting due to (a) platelet dysfunction, (b) renal impairment, (c) gastrointestinal disorders and (d) bleeding complications [15]. There are concerns especially with their effects on platelets which have limited their use in the immediate preoperative period [16]. The development of COX-2-selective agents has provided additional options for the management of acute pain. COX-2-inhibitors may offer benefits in the pre- and perioperative settings because of their selective inhibition of COX-2. Several studies report significantly lowered postoperative pain scores such as a significantly reduced dose of postoperative opioids [17-19]. In addition, their unique pharmacologic profile makes them a promising alternative to NSAIDs [20].

To our knowledge, there are only a limited number of publications examining the preemptive effect of COX-2-inhibitors on the intensity of postoperative pain. All these studies deal with orthopaedic surgery [21-23]. We therefore carried out a novel study design to examine these preemptive and postoperative analgetic effects of COX-2-inhibitors, in particular etoricoxib, in patients programmed for abdominal or thoracic surgery. In addition, we aimed to follow-up the occurrence of persistent pain.

## Patients and Methods

### Study Design

The study is a 2 × 2 factorial armed, double blinded, randomised placebo-controlled trial comparing (a) etoricoxib and (b) placebo in a pre- and postoperative setting. After randomisation, patients receive preemptive medication of either (a) or (b). Medication will be given postoperatively for additional three days. Additionally, all patients receive morphine administered through Patient Controlled Analgesia (PCA). Analysis of all records is performed by blinded evaluators. The total follow-up period per patient is 6 month. Trial registration is NCT00716833.

### Patients

For the included patients the following criteria must be met:

- Age ≥ 18 years

- Scheduled abdominal or thoracic surgery harming peritoneum or pleura

- ASA classification I or II according to the American Society of Anaesthesiologists

Main exclusion criteria are:

- Severe cardiac/pulmonary/renal or neurologic diseases

- ASA score > II

- Patients with insulin-dependent diabetes mellitus or other diseases influencing the peripheral sensibility (e.g. polyneuropathy, chronic pain syndromes)

- Regional blocks

- Continuous use of analgesics

- Pregnancy or lactation

- Uncontrolled hypertension

- Contraindications listed in the product information of etoricoxib, i.e. intolerance, ulcers or gastric bleeding, inflammatory bowel diseases, anaphylactic reactions

Information will be held on the vigil of surgery. If criteria are appropriate and patients want to participate, they give their written informed consent with sufficient time of consideration.

### Randomised treatment allocation, blinding and sample-size estimation

**Figure 1:**
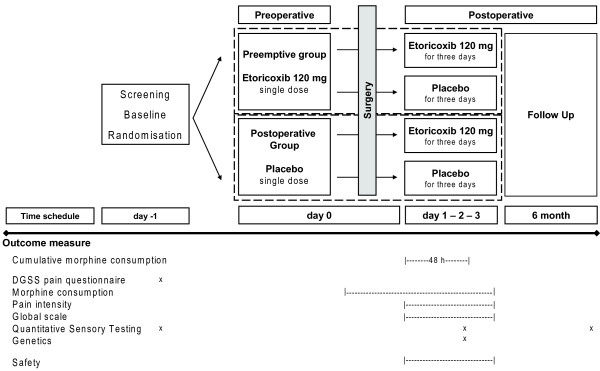
**Study Design**. Patients are randomly allocated to either the preemptive or the postoperative treatment group. Preemptive group patients get etoricoxib 120 mg p.o. prior to surgery (day 0, preoperative). After surgical intervention, depending on their respective allocation, patients receive either etoricoxib 120 mg p.o. or placebo for the next three postoperative days (day 1-2-3, postoperative). Postoperative group patients get placebo prior to surgery and continue afterwards with either placebo or etoricoxib 120 mg p.o. for three postoperative days (2 × 2 factorial study design). The listed outcome measures will be assessed at the marked time points respectively.

Patients are randomly allocated to either the preemptive or the postoperative treatment group. These two groups are each divided into two arms. Preemptive group patients receive etoricoxib prior to surgery and either etoricoxib again or placebo postoperatively. Postoperative group patients receive placebo prior to surgery and either placebo again or etoricoxib after surgery (2 × 2 factorial study design, Figure 1). The randomisation procedure into the two study arms was performed by the Institute of Medical Information Technology, Biometry and Epidemiology, University of Munich, Germany. The biometricians compiled an allocation list which was the basis for the pharmacists to prepare sequentially numbered envelopes containing two boxes of study medication for pre- and postoperative use. The boxes contained either a 120 mg dose of etoricoxibe or an equivalent placebo pill. After inclusion into the trial, the study physician assigned the content of the lowest numbered envelope to the patient. Neither the patient nor the study physician knew about the content of the boxes, the pills were not distinguishable. This procedure assured a complete blinding.

We conducted a 2 × 2 factorial-armed study which should prove the superiority of preemptive versus postoperative etoricoxib. The study was based on the moderate effective size of pre-emptive etoricoxib vs. post-operative etoricoxib in the reduction of the 48 hour cumulative morphine consumption. A sample size of approximately 60 patients in the two arms (i.e. 30 patients in each of the four arms) is anticipated taking a drop out rate of 10% into account (this is a conservative estimate based on the dropped out rate of 4% in the study by Sinatra et al. [19]). With this sample size we would permit a type I error of alpha = .05, and with the alternate hypothesis, the null hypothesis would be retained with a type II error of beta = .2 (i.e. power of 80%).

The calculation of the effective size is based on the data of Sinatra et al. [19]. The authors found a mean reduction of cumulative morphine consumption of 23 mg in the treatment group (total morphine dose 45 mg, SD 14) compared to the placebo group (total morphine dose 68 mg, SD 25).

### Participating Physicians

Participating trial physicians are employees of the Multidisciplinary Pain Centre, Department of Anaesthesiology, University of Munich, Germany. Their average qualification is at least equal to a 3^rd ^year resident in the field of anaesthesiology and specialised pain medicine. They contributed to all medical duties.

### Interventions

Patients are randomly allocated to either the preemptive or the postoperative treatment group. Preemptive group patients get etoricoxib 120 mg p.o. prior to surgery (day 0). After surgical intervention, depending on their respective allocation, patients receive either etoricoxib 120 mg p.o. or placebo for the next three postoperative days (day 1-3). Postoperative group patients get placebo prior to surgery and continue afterwards with either placebo or etoricoxib 120 mg p.o. for three postoperative days (2 × 2 factorial study design, Figure 1). All patients will receive standardised morphine (patient controlled analgesia PCA; bolus 2 mg; interval 10 min; max dosage 30 mg/4 h). Patients, study physicians and contributing hospital staff are blinded regarding the treatment group allocation.

### Ethics

The study is performed according to the principles of the Declaration of Helsinki (Version Edinburg 2000, cf. http://www.wma.net/en/30publications/10policies/b3/index.html) and according to common guidelines for clinical trials (ICH-GCP). The protocols have been approved by the Ethics Committee, University of Munich and the national component authority (German Federal Drug Administration [Bundesinstitut für Arzneimittel und Medizinprodukte BfArM]). Written informed consent is obtained from all patients.

### Outcome measures

#### Main outcome measure

The cumulative morphine consumption (mg) within the first 48 hours after surgery (PCA)

#### Secondary outcome measure

- Baseline personal situation on the DGSS (German Society for the Study of Pain) validated pain questionnaire [24], comprising inter alia the German versions of questionnaires assessing demographic data, pain variables (e. g. pain sites, temporal characteristics, duration, intensity), pain associated symptoms, affective and sensory qualities of pain (adjective list by Geissner, SES), pain relieving and intensifying factors, previous pain treatment procedures, pain-related disability (Pain Disability Index), depression (Centre for Epidemiological Studies Depression Test CES-D), comorbid conditions, social factors (educational level, occupation, retirement status, compensation or litigation status, disability for work), health related quality of life (SF-36).

- Systematic quantitative sensory testing (QST). The detailed QST protocol including reference data is reported elsewhere [25]. In brief, the following testing procedures were performed:

a) **Thermal Testing **comprising cold and warm detection thresholds (CDT, WDT), paradoxical heat sensations (PHS) during the thermal sensory limen procedure (TSL) of alternating warm and cold stimuli and cold and heat pain thresholds (CPT, HPT);

b) **Mechanical Testing **comprising mechanical detection thresholds (MDT), mechanical pain thresholds (MPT), mechanical pain sensitivity (MPS), dynamic mechanical allodynia (DMA), the wind-up ratio (WUR), vibration detection thresholds (VDT) and pressure pain thresholds (PPT).

- Pain intensity (visual analogue scale)

- Overall morphine consumption

- Side effects (documentation according to ICH - GCP Guidelines)

- Genetic polymorphisms: the metabolic profile of etoricoxib involves cytochrome P450-dependent hydroxylation and oxidation as primary clearance pathways [26]. Different genotypes might either be the reason for individually different analgesic needs or be considered as a risk factor for side effects [27]. Similar mechanisms have been described for the use of opioids and so called multidrug resistance proteins (MDR) [28]. We therefore collected EDTA-blood samples from all patients to analyse the different expression of genotypes.

For time points please refer to Figure 1.

### Data Analysis

Descriptive analysis of the study population (including means, standard deviations, median and frequencies) will be made for all parameters.

Main outcome measures will be analysed with a 2 × 2 factorial ANOVA. Independent variables are time point of medication uptake and allocated study arm, dependent variable is the cumulative morphine consumption within 48 hours postoperatively.

Secondary outcome measures (QST, pain intensity) will be analysed equivalent, taking into account the test interval (repeated measurement ANOVA). ANOVA will be adjusted according to Bonferroni using t-test for post-hoc confirmation of significant alterations.

Ordinal scaled data will be analysed with Kruskal Wallis test confirming significance with chi2 test.

For analyses of associative coherence we will use Spearman's correlations coefficient, Wilcoxon-test or Kruskal Wallis test. All reports will be performed according intention-to treat analysis.

### Data entry

Data entry is done with SPSS statistical software system (SPSS Inc., Chicago, IL; version 15.0). Data analysis will be done with SAS/STAT^® ^Software (SAS Institute Inc., Cary, NC, USA). All data entry will be carried out twice.

### Monitoring

Internal and external audits will be held in order to assure quality standards according to ICH-GCP guidelines, the Declaration of Helsinki and governmental standards.

## Discussion

To our knowledge, the EPPA trial is the first clinical study to investigate the preemptive analgesic effect of etoricoxib on the cumulative postoperative morphine consumption applying a 2 × 2 factorial study design. In addition, our protocol takes into account individual sensoric perception of the skin as well as genetically different polymorphisms regarding the drug action.

Inclusion and exclusion criteria were deployed pragmatically in order to facilitate screening and recruitment. Exclusion criteria (besides standard items such as pregnancy or contraindications to the study medication) are either disease interfering with the patients' sensory perception or with expected side effects or possible harm related to etoricoxib. Our inclusion and exclusion criteria are based on further trials and according to the summary of product information [29,30]

## Etoricoxib

Several COX-2-inhibitors have been shown to be effective treating postoperative pain [31-33]. Data for the use of etoricoxib seem to be promising; however, existing results describing preemptive and postoperative effects remain heterogeneous [34-36]. Etoricoxib is a COX-2-selective NSAID which is approached for treatment of osteoarthritis, rheumatoid arthritis, acute gouty arthritis and Morbus Bechterew. Numerous studies indicated that etoricoxib has similar efficacy as traditional, unselective NSAIDs have. It does not seem to elevate the risk of severe side effects, i.e. in special vascular events [29,37]. The rates of thrombotic cardiovascular events in 34.000 patients with arthritis on etoricoxib were similar to those in patients with long-term use of diclofenac [29]. Ex vivo whole-blood assays after multiple oral doses of etoricoxib showed no important effects on bleeding time or platelet aggregation [38]. Consequently, these drugs do not carry the risk of blocking surgical interventions.

Additionally, etoricoxib demonstrated superior safety in gastrointestinal toxicity due to its high selective COX-2 inhibition that is observed with its use. Other NSAID-associated effects, including renal adverse effects, appear to be similar to those of other traditional NSAIDs [20].

The pharmacokinetic evaluation of etoricoxib indicates a moderate rate of absorption and a t1/2 of approximately 20 hours that enables once-daily dosing [20].

We have chosen etoricoxib as verum treatment due to the presented pharmacologic properties such as our own clinical experience dealing with. Drug approval for peri- and postoperative setting has not yet been authorised.

## Quantitative sensory testing

Postoperative pain involves not only peripheral mechanisms, most notably the sensitization of nociceptors due to inflammation, but also secondary central mechanisms, including hyperexcitability of nociceptive neurons (i.e., central sensitization, [39,40]). These processes play a major role in postoperative pain, including spontaneous pain and allodynia or hyperalgesia. In particular, peripheral sensitization would explain the hyperalgesia observed at the incision site (primary hyperalgesia), whereas central sensitization would provide a major mechanism of secondary hyperalgesia at distant noninflammatory sites [41-43]; thus indicating a possible pathway for the occurrence of prevalent pain.

From a clinical perspective, preoperative measurement of sensoric perception may have some predictive value regarding postoperative pain and, therefore, may also predict perioperative analgesic requirement [44]. Martinez et al. showed that preoperative heat hyperalgesia directly correlated with postoperative morphine consumption after total knee arthroplasty [43]. These results are in agreement with those of previous studies on the prognostic value of preoperative pain for immediate postoperative pain intensity with other types of surgery [45,46]. Postoperative segmental secondary hyperalgesia was detected in patients undergoing different types of surgical interventions [47-49]. Several central and peripheral pathophysiological actions play a role in the development of acute and chronic postoperative pain. Quantitative sensory testing (QST) allows precise characterization of sensory deficits and painful symptoms and may offer additional information on the pathophysiology of postoperative pain. A follow-up sensory testing in our trial is performed six months after intervention. Therefore, we will be able to provide information regarding long-term sensitisation after surgery, too. Taken together, we will be able to evaluate possible predictive factors related to patients' individual sensoric perception that may influence the intensity of postoperative pain and analgesic consumption such as characteristics that might predict or prevent chronification.

## Genetics

Genetic polymorphisms in P-glycoprotein (P-gp), a membrane-localised transporter codified by the gene MDR1 and Cytochrome P450 (CYP) genes, are involved in drug metabolism and often account for variable drug response or side effects. Some common drugs, including non-steroidal anti-inflammatory drugs, are metabolised by the P450 CYP2 C9 enzyme. There are mostly three variants of CYP2C9 gene which show alternated drug response. The variants CYP2C9*1, *2 and *3 occur most frequently with those of a caucasian background [50]. The enzymatic activity of *2 and *3 variant genes is decreased significantly, and carriers of the *3 variant are at risk for complications, such as bleeding after use of warfarin in small amounts [51]. Newer COX-2 inhibitors have less potential for causing gastrointestinal bleeding. The COX2 inhibitor etoricoxib has plasma protein binding of about 92% and is extensively metabolised, with only about 1% being excreted in urine as parent drug. CYP3A4 plays a major role in the metabolism of etoricoxib (60%), and CYP2C9, CYP2C19, CYP2D6 account for only a minor fraction ( 10%) of etoricoxib's metabolic profile [52]. Even though the pharmacokinetic profile is linear and in vitro studies support that etoricoxib does minor induction or inhibition of CYP isoenzymes, we want to test whether the mentioned CYPs or the polymorphisms of the MDR1 (e.g. C3435T) have a possible connection with the outcome of postoperative pain management.

## Study Design

We have chosen a 2 × 2 factorial design for the following reasons: the factorial design has several important features. Firstly, it has great flexibility for exploring the treatment effects in the trials. Whenever examining treatment variations, factorial designs are strong candidates as the design of choice. Secondly, factorial designs are efficient due to the ability to combine multiple studies into one rather than conducting a series of independent studies. Finally, factorial designs are the only effective way to examine interaction effects [53,54].

If the primary aim of a trial is to identify useful single treatments, then it could possibly be more cost effective to use a three-arm study than a 2 × 2 factorial design. In this trial, it is of interest to detect the treatment interaction, i.e. the opioid-sparing effect of a non-opioid treatment. The chosen study design has the power to identify such statistically significant differences between etoricoxibe and placebo treatments [55]. While the methods are developed for binary outcomes, they can be readily adapted to outcomes based on continuous, ordinal or time-to-event data using the methods for power calculation [55,56]. No attempt has been made to adjust for multiple comparisons. However, this can be achieved by simply changing the level of significance according to some recognised procedures such as the Bonferroni correction [55,57]. In our opinion, the study design allows to obtain specific results regarding the preemptive and postoperative analgesic effects of etoricoxib on (a) the cumulative morphine consumption and (b) other parameters as mentioned: predisposing characteristics influencing postoperative pain (inter alia by means of questionnaires), sensoric perception, genetic polymorphisms and others.

## Conclusion

This study is a large-scale randomised placebo-controlled trial to evaluate the efficacy of COX-2-inhibitors in preemptive and postsurgical pain therapy. It can be expected to provide new valuable information on clinical and pathophysiological effects in postoperative pain, i.e. a) the analgetic effectiveness of preemptive analgesia and b) its relation to e.g. sensoric perception or genetical predisposition as possible factors leading to persistent (chronic) pain.

## Competing interests

All authors declare that they have no competing interests and did not receive any honorarium from MSD Sharp and Dome or other partners. The investigator-initiated grant received by MSD Sharp and Dome guarantees independent conceivability of the study design, its coordination, realisation and independent report of the study results.

## Authors' contributions

JF participated in the study design, patient recruitment and trial coordination, and drafted the manuscript. SK, LL and PML conceived of the workflow and acted as trial physicians. BH, HP, PC, MS, TM, PML and GS participated in the design of the study. PC, MS and TM coordinated the study in their departments. MO performed the sample size estimation and conceived of the biometrical study design. DI is the principal investigator and initiator of the study, obtained funding, designed the study and supervised and participated in writing the manuscript. All authors read, and approved the final manuscript.
